# A comparative study on eDNA-based detection of Siamese bat catfish (*Oreoglanis* *siamensis*) in wet and dry conditions

**DOI:** 10.1038/s41598-024-58752-x

**Published:** 2024-04-17

**Authors:** Maslin Osathanunkul, Chatmongkon Suwannapoom

**Affiliations:** 1 Department of Biology, Faculty of Science, Chiang Mai University, Muang District, Chiang Mai, Thailand; 2 School of Agriculture and Natural Resources, University of Phayao, Muang District, Phayao, Thailand

**Keywords:** eDNA analysis, Endemic species, Freshwater species detection, Rainfall influence, Siamese bat catfish, Ecology, Molecular biology, Ecology, Environmental sciences

## Abstract

The use of environmental DNA (eDNA) analysis has demonstrated notable efficacy in detecting the existence of freshwater species, including those that are endangered or uncommon. This application holds significant potential for enhancing environmental monitoring and management efforts. However, the efficacy of eDNA-based detection relies on several factors. In this study, we assessed the impact of rainfall on the detection of eDNA for the Siamese bat catfish (*Oreoglanis siamensis*). Quantitative polymerase chain reaction (qPCR) analysis indicated that samples from days with average rainfall exceeding 35 mm (classified as heavy and very heavy rain) yielded negative results. While eDNA detection remains feasible on light or moderate rainy days, a noteworthy reduction in eDNA concentration and qPCR-positive likelihood was observed. Analysis across 12 sampling sites established a statistically significant negative relationship (*p* < 0.001) between eDNA detection and rainfall. Specifically, for each 1 mm increase in rainfall, there was an observed drop in eDNA concentration of 0.19 copies/mL (±0.14). The findings of this study provide definitive evidence that precipitation has a significant impact on the detection of eDNA in Siamese bat catfish. However, in the case of adverse weather conditions occurring on the day of sampling, our research indicates that it is acceptable to continue with the task, as long as the rainfall is not heavy or very heavy. To enhance the effectiveness of an eDNA survey, it is crucial to consider many factors related to climatic conditions. The aforementioned factor holds significant importance not only for the specific species under scrutiny but also for the broader dynamics of the climate.

## Introduction

In recent years, environmental DNA (eDNA) detection has acquired popularity as a potent tool for surveying and monitoring biodiversity in diverse ecosystems. The eDNA technique requires the collection and analysis of genetic material shed by organisms into their surrounding environment, such as water or sediment. This genetic material can be extracted and examined to determine the presence of particular species or communities. In addition to its sensitivity, eDNA detection has also been shown to be more effective than traditional survey and monitoring approaches (e.g.,^1–7^). Traditional methods often rely on direct observation or trapping of organisms, which can be time-consuming, labour-intensive, and may not capture the full range of species present in an ecosystem. In contrast, eDNA analysis can provide a comprehensive snapshot of biodiversity in a given area, allowing for a more accurate assessment of species richness and community composition. Initial investigations suggest that eDNA techniques have higher probability to detect rare species^8^. eDNA has been found to provide a higher resolution for detecting species than conventional methods such as fyke net catches and electrofishing^5,6^. In addition, eDNA analysis is a nondisruptive method which reduces the need for repeated visits and labour-intensive sampling efforts associated with traditional methods^9–11^. The cost-effectiveness of eDNA detection is particularly evident when examining a large number of sites, as eDNA sampling has lower field labour and transportation costs compared to traditional sampling^12–14^. Overall, the evidence suggests that eDNA detection is not only more sensitive but also more effective than traditional capture-based surveys.

Future use of eDNA detection is likely to increase as technology continues to advance, contributing to our understanding and management of the natural world. Nonetheless, a number of biotic and abiotic factors have been shown to influence the detection of eDNA. Biotic factors refer to the living organisms present in the environment, while abiotic factors encompass the non-living components such as temperature, pH, salinity, and hydrological processes^15–20^. Biotic factors include the presence of extracellular enzymes and microorganisms, which can influence eDNA decay^21^. Additionally, factors like currents, temperature, and species abundance patterns can influence both eDNA and visual detection^22^.

Several studies have investigated the effect of rainfall on the detection of eDNA in diverse aquatic and terrestrial environments. In aquatic systems, high water flow, heavy rain, clay particles, and water turbidity can reduce the likelihood of eDNA detection^23^. Local factors like rainfall can affect the transport and detection of eDNA in rivers, potentially leading to variations in detection distance and time^15^. Furthermore, climate data, including rainfall, are important in eDNA surveys and data interpretation as rainfall can dilute eDNA concentration and reduce detection probabilities, highlighting the need for repeated sampling and collection of abiotic variables like rainfall^24^. Aucone et al.^25^ also observed a decrease in eDNA detection in relation to significant heavy rainfall. They suggest that rainfall can wash away eDNA present on vegetation surfaces, impacting surface detection and the transport and fate of eDNA on aboveground substrates. In addition, Villacorta-Rath et al.^26^ hypothesise that rainfall can affect the detectability of eDNA by increasing water flow and diluting it. According to Johnsen et al.^23^, high water flow, heavy rain, clay particles, and water turbidity can reduce the likelihood of eDNA detection. Interestingly, rainfall has been found to limit the detection of airborne eDNA particles in terrestrial environments^27^. In contrast, Staley et al.^28^ discovered that an extreme rainfall event altered the eDNA profiles of creek and beach sites, resulting in an increased diversity of mammal and bird eDNA sequences.

The focus here lies on the influence of rainfall amounts on the detection of environmental DNA (eDNA). Forecasting the occurrence of rain on the day of collecting an eDNA sample can be difficult. The sampling was frequently premeditated several months ahead of time and encompassed a substantial number of individuals. While previous research has shown that rainfall can impact the detection of environmental DNA (eDNA), our objective was to determine the threshold of rainfall intensity (ranging from trace to very heavy rain) at which eDNA detection would become unreliable, leading to unsuccessful sample collection.

## Results

### qPCR assay and inhibition test

Limit of detection (LOD), and the limit of quantification (LOQ) was calculated and found to be 5.75 copies/reactions for both values. The Cq cut-off value for positive sample was 39 cycles. All qPCR results were reported into three categories which are (1) positive with quantifiable eDNA concentration, below limit of quantification (bq: Cq = 39.01–44.99), and non-detect (nd: Cq ≥ 45 or No amplification). All the assays without the internal control templates showed negative amplification. The average of ΔCq values from the internal controls of all samples were less than 3, which lower than the inhibition criteria (Cq shift of ≥ 3 cycles was an indication of inhibition). Therefore, PCR inhibition was not likely to occur in all samples.

### Rainfall and eDNA detection

Surface waters at twelve sampling sites where presence of the target species known were collected on the day with rain and no rain at all. Rainfall occurred in the two to three days leading up to the rainy-day sampling, resulting in elevated water levels and increased turbidity on the day of sample collection. Furthermore, under typical sunny conditions, water samples exhibit clarity, but on rainy days, they manifest a murky brown appearance, posing challenges during the filtration process. Additionally, heightened water flow in the stream, as depicted in Supplementary Fig. 1, was observed during the rainy-day samplings. Despite the difficulty, 900 mL of water samples was filtered, the colour of filter papers was also found to be brown to dark brown comparing with light yellow when filtering on the non-rainy days (Supplementary Fig. 2). The DNA was extracted from the filtered papers and undergone the qPCR assay.

**Figure 1:**
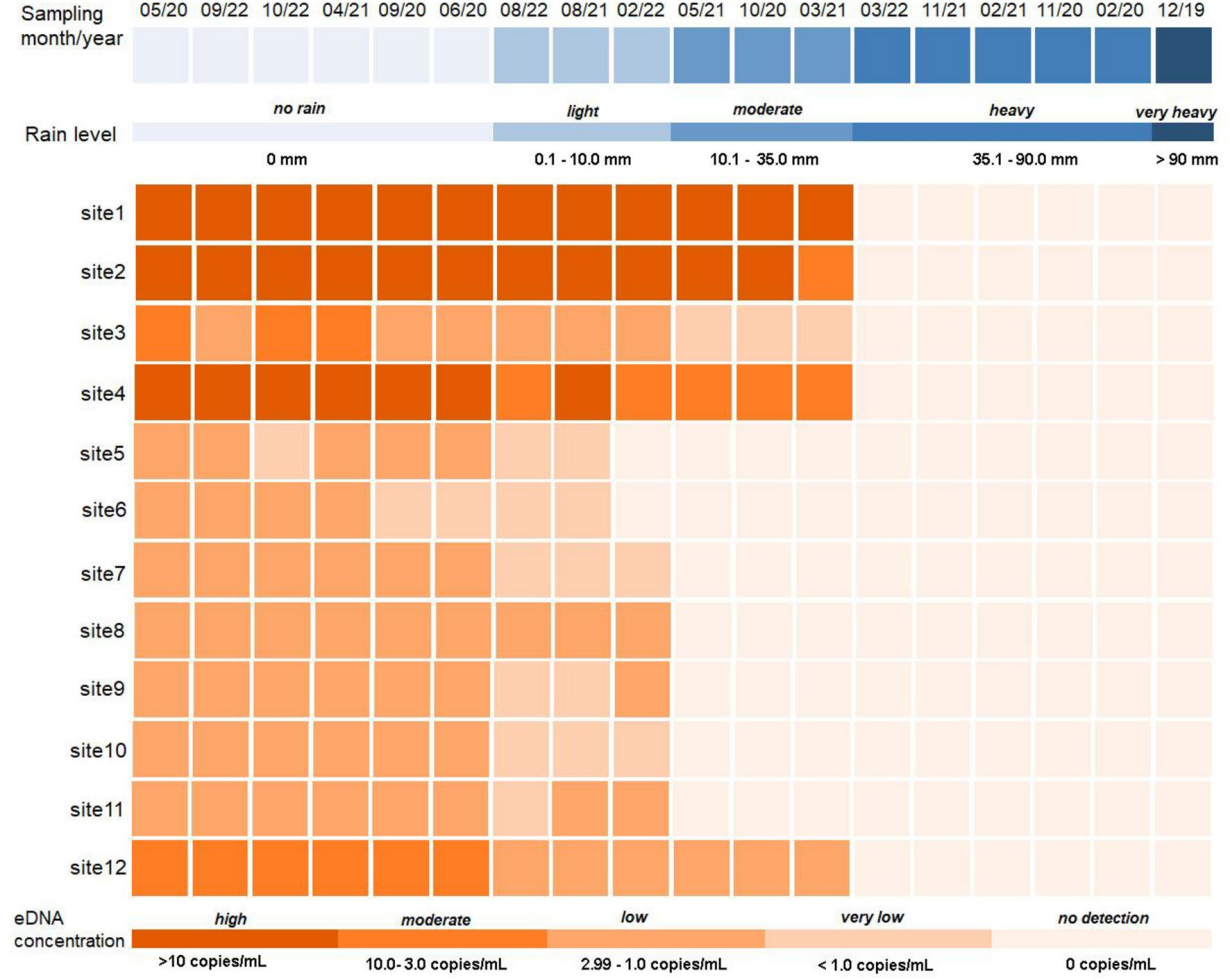
eDNA concentration of Siamese bat catfish detected at each site on every sampling day, along with the corresponding rainfall data.

The presence of Siamese bat catfish eDNA was observed in the samples obtained during days without rainfall. The concentration of copies per millilitre at each site was documented and presented in Fig. 1 and Supplementary Table 1. Siamese bat catfish eDNA was discovered in all twelve collection sites on days without rainfall, exhibiting varying quantities. The concentration of eDNA at each sampling site remained consistent across the six non-rainy days of sampling. The average concentration of eDNA was found to be 20.56 copies/mL (SD = 0.34), 17.66 copies/mL (SD = 0.25), 2.45 copies/mL (SD = 1.00), 12.18 copies/mL (SD = 0.23), 1.39 copies/mL (SD = 0.29), 1.36 copies/mL (SD = 0.48), 1.14 copies/mL (SD = 0.08), 2.26 copies/mL (SD = 0.11), 2.04 copies/mL (SD = 0.12), 2.12 copies/mL (SD = 0.07), 2.15 copies/mL (SD = 0.08), and 4.15 copies/mL (SD = 0.11) for sites 1–12, respectively. In contrast, the samples taken on days with heavy and very heavy rainfall did not provide any detection of Siamese bat catfish eDNA, even at the same places where positive results were obtained on non-rainy days (Fig. 1). It is worth noting that the eDNA of the Siamese bat catfish was measured in water samples obtained during days with moderate rainfall at certain locations (sites 1–4 and 12). The presence of eDNA was observed at all sample locations during days with light rain, with the exception of site 6 on the specified sampling date (Supplementary Table 1).

**Figure 2:**
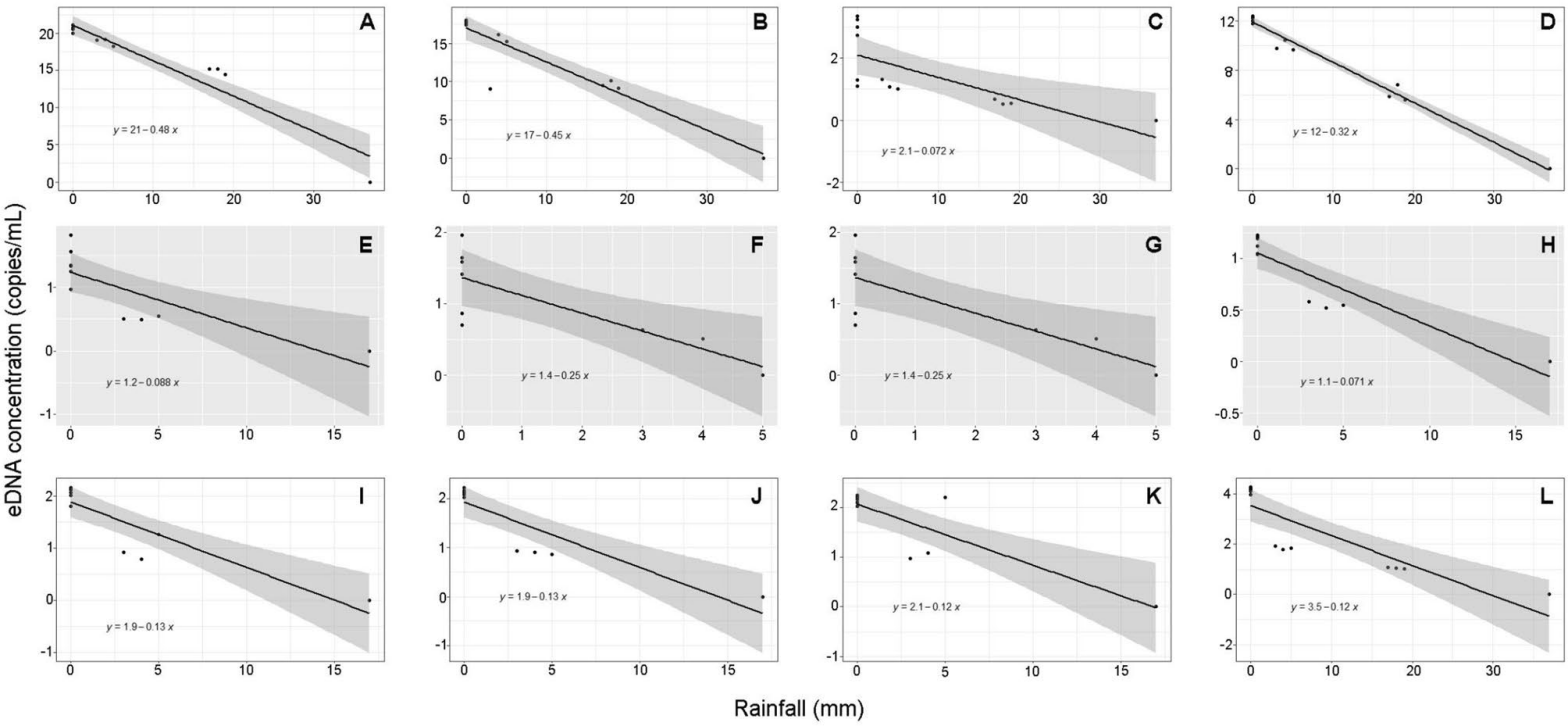
Plotted data graphs obtained from regression analysis, which were used to assess the correlation between the concentration of eDNA measured in copies per millilitre (copies/mL) and the amount of rainfall recorded in millimetres (mm). The outcomes of site 1 to 12 are represented by the letters A to L, respectively. In each graph, a linear regression line has been incorporated, accompanied by the inclusion of a light grey stripe encircling the line, which represents the standard error of the estimate.

The analysis focused on examining the link between the occurrence of rainfall and the detection of eDNA across all twelve designated sampling sites. The findings depicted in Fig. 2A–L demonstrate a statistically significant correlation between rainfall and eDNA detection (*p* < 0.0001). The largest coefficient of determination (*R*^2^) was observed at site 4, reaching a value of 0.98, while the least *R*^2^ was observed at site 10, with a value of 0.80. The study revealed a statistically significant inverse correlation, indicating that for each 1 mm rise in rainfall, there was a decrease of 0.19 copies/mL (± 0.14) in eDNA concentration (Supplementary Table 2).

## Discussion

### Rainfall and eDNA detection

The detection of eDNA is subject to the influence of both biotic and abiotic variables. Biotic factors pertain to the organisms that inhabit a given environment, whereas abiotic factors encompass the non-living constituents, including temperature, pH, salinity, and hydrological processes^15–20^. The rainfall effect is the main focal factor in this study. Rainfall can have both direct and indirect effects on eDNA detection. One of the primary ways rainfall influences eDNA detection is through dilution. Heavy rainfall events can result in increased water volumes and subsequent dilution of eDNA present in the sampled environment. This dilution effect reduces the concentration of target DNA, potentially making it more challenging to detect low-abundance species or detectable genetic signals^29,30^. Dilution effects can vary depending on the intensity and duration of the rainfall event, as well as the hydrological characteristics of the sampling site.

Rainfall also plays a role in the transport and dispersal of eDNA. During rainfall, water can carry eDNA through runoff and streams, causing the genetic material to be transported beyond the sampling area. This dispersion of eDNA can lead to a loss of localised genetic signals, making it more difficult to detect specific species or organisms of interest^16,31^. Additionally, rainfall-induced dispersal can complicate the interpretation of eDNA results, as the detected DNA may originate from sources outside the immediate sampling site. The degradation of eDNA is another significant factor influenced by rainfall. Precipitation may introduce various agents into water samples that could impact DNA detection, though literature evaluating these dynamics is limited. Heavy rainfall can indirectly accelerate DNA degradation, reducing its detectability and potentially shortening its persistence in the environment^32^. The extent of degradation depends on rainfall characteristics, environmental conditions, and the stability of the eDNA target region. Furthermore, rainfall can impact eDNA detection through the filtration and trapping methods used during sample collection. Heavy precipitation increases turbidity and particulate matter loading in aquatic ecosystems. This turbid water quality in turn causes the eDNA sampling filtrate to become laden with debris that can overwhelm filters, impeding their ability to effectively capture eDNA from water samples^33,34^. Adjustments in sampling protocols, such as modifying filtration techniques or using alternative collection methods, may be necessary to mitigate the effects of rainfall on eDNA capture efficiency.

The results of this study, which used Siamese bat catfish as a model fish and collected samples in Thailand, show a strong negative relationship between the amount of eDNA found and the amount of rain. The eDNA of the target species was shown to be detectable on days with rainfall, but at a reduced concentration, as indicated in Fig. 1 and Supplementary Table 1. Nevertheless, the initial concentration of eDNA varied when sampling was conducted on non-rainy days, resulting in variations in the extent of reduction and detection efficacy across different categories of rainy days, such as light rain, moderate rain, and heavy rain. The majority of eDNA from Siamese bat catfish identified at the sampling sites exhibited modest concentrations, as outlined in the criteria described in the Methods section. The Siamese bat catfish exhibited a high concentration of eDNA at sampling sites 1, 2, and 4, whereas a moderate concentration was detected at sites 3 and 12. The study of correlations between locations reveals a strong association between the initial concentration of eDNA and the degree of rainfall. Specifically, the results indicate that a 1 mm increase in rainfall is associated with a reduction of around 0.5 copies/mL of a high level of initial Siamese bat catfish eDNA (as shown in Supplementary Table 2). In contrast to the low and medium levels of initial eDNA, it has been observed that the measured eDNA decreases by up to 0.14 copies/mL for every 1 mm increase in rainfall. When the results from all sites were combined, it was seen that there was a drop in eDNA concentration of 0.19 copies/mL (± 0.14) with every 1 mm increase in rainfall. This is believed to be an outcome of the phenomenon of rainfall dilution, as previously mentioned. Furthermore, it was discovered that water velocity at the sampling sites increased during rainy days, as depicted in Supplementary Fig. 1. Consequently, it is probable that the eDNA present at the sampling location was transferred downstream.

### Recommendations for tropical eDNA studies

The prediction of rainfall, particularly in Thailand, is a significant challenge. In certain instances where sampling dates are fixed and non-negotiable, it may be prudent to take meteorological conditions into account. Based on the findings presented herein, it is reasonable to consider cancelling the sampling activity in the event of heavy or very heavy rainfall (rain more than 35 mm) on the designated date. In the event of a light rainfall and the availability of past eDNA data indicating moderate or high eDNA concentrations of the target species at the designated sites, it is feasible to proceed with the collection of water samples. In conclusion, it is imperative to acknowledge that the impact of precipitation on the detection of eDNA can exhibit variability across diverse ecosystems, species, and sampling methodologies. The complexity of this relationship is influenced by various factors, including the attributes of the target organism, rates of eDNA degradation, hydrological dynamics, and local patterns of rainfall. Hence, it is imperative to possess a thorough comprehension of these parameters in order to develop resilient eDNA sample techniques and appropriately interpret outcomes within the framework of rainfall effects.

## Methods

### Sampling sites and eDNA collection

The Mae Klang Phat and Mae Klang rivers in Doi Inthanon National Park, Chiang Mai, Thailand, were selected for field validation of the Siamese bat catfish (*Oreoglanis siamensis* Smith, 1993) eDNA assay. The current study evaluates rainfall effects across an appropriate subset of sites where Siamese bat catfish eDNA occurred based on prior data^35^ to examine the impact of rainfall intensity on the detection of Siamese bat catfish eDNA (Supplementary Table 3).

Water samples were collected in triplicate (sample1–sample3) at the same location, on non-rainy days six times, and on rainy days, twelve times (Table 1). The samples were gathered in accordance with Rodpai et al.^35^, surface water samples were collected in sterile pails (each pail was disinfected with 10% bleach and then rinsed twice with distilled water). 300 mL of water sample were filtered in the field using Whatman glass fibre filters (pore size: 0.7 µm) in filter housings connected to BD Luer-LokTM 50 mL syringes. At each site, a multi-filter was collected (3 filters of 300 mL each or 9 filters of 100 mL each on rainy days), with a total of 900 mL of water used for filtration to constitute a single sample replicate. One negative field control of 900 mL ddH_2_O was also filtered per location. At each location, this protocol was executed with 3 samples and 1 negative control and then filters were placed in 1.5 mL microcentrifuge containers using tweezers. To prevent cross-contamination, the tweezers were rinsed with 10% bleach and 70% ethanol, and the mitts were replaced between each sample. All cylinders were stored in a polystyrene box containing dry ice until they were transferred to − 20 °C.

**Table 1:** Average rainfall of each sampling day.

Sampling date	Average rainfall (mm)	Category
14 Dec 19	0	Non-rainy day
10 Feb 20	0	Non-rainy day
28 Jun 20	37	Rainy day/*heavy* ***
9 Sep 20	46	Rainy day/*heavy* ***
4 Oct 20	4	Rainy day/*light* *
22 Nov 20	0	non-rainy day
6 Feb 21	0	non-rainy day
23 Mar 21	3	rainy day/*light* *
6 Apr 21	56	Rainy day/*heavy* ***
10 May 21	5	Rainy day/*light* *
28 Aug 21	18	rainy day/*moderate* **
19 Nov 21	0	non-rainy day
20 Feb 22	17	Rainy day/*moderate* **
2 Mar 22	0	non-rainy day
21 May 22	128	rainy day/*very heavy* ****
27 Aug 22	19	Rainy day/*moderate* **
10 Sep 22	88	Rainy day/*heavy* ***
1 Oct 22	76	Rainy day/*heavy* ***

Rainfall was categorised into five categories according to Thai Meteorological Department criteria, as follows: (#) *trace* is when rainfall volume is less than 0.1 mm; (*) *light rain* is 0.1–10.0 mm; (**) *moderate rain* is 10.1–35.0 mm; (***) *heavy rain* is 35.1–90.0 mm; and (****) *very heavy rain* is when it exceeds 90.1 mm.

### eDNA extraction

Within 48 h after sampling, DNA was extracted using a DNeasy Blood and Tissue Kit (Qiagen, Hilden, Germany) with a slight modification to the protocol from the manufacturer’s following the Osathanunkul & Minamoto^29,30^. Each extracted DNA sample was then treated with the OneStep PCR Inhibitor Removal Kit (Zymo Research) to remove potential PCR inhibitors prior stored at − 20 °C until further processing.

### qPCR assay and sensitivity testing

All tissue samples of Siamese bat catfish were provided by the Department of Fisheries, Thailand. Total DNA was extracted from the tissue sample using the DNeasy Blood and Tissue Kit (Qiagen, Hilden, Germany). Extracted DNA was used as a template for assay validation. Species-specific primers for Siamese bat catfish (forward primer: 5′-CCTTGCAGGTGTATCGTCTATTC-3′ and reverse primer: 5′-AGCTGCCAAGACTGGTAGT-3′) and 5′-6-FAM labelled TaqMan® minor groove binding probe (5′-CCTCCAGCAATTTCCCAATACCAAACC-3′) targeting a portion of the mitochondrial cytochrome oxidase I (COI) gene were designed using PRIMER EXPRESS 3.0 (Applied Biosystems-Roche, Branchburg, NJ) with total amplicon size of 160 bp (based on DNA sequences shown in Supplementary Table 4). Probe and primers were matched against the nucleotide database with BLASTn (Basic Local Alignment Search Tool) to confirm the species specificity for Siamese bat catfish. The primers were also validated on DNA of Siamese bat catfish, closely related fish species (*Oreoglanis colurus* Vidthayanon, Saenjundaeng & Ng, 2009 and *Oreoglanis vicina* Vidthayanon, Saenjundaeng & Ng, 2009) and co-occurring non-target species including *Barbonymus gonionotus* (Bleeker, 1849)*, Channa aurolineatus* (Cuvier, 1831)*, C. micropeltes* (Cuvier, 1831)*, C. striata* (Bloch, 1793)*, Chitala ornate* (Gray, 1831)*, Garra cambodgiensis* (Tirant, 1883)*, Hypsibarbus malcolmi* (Smith, 1945)*, Labiobarbus siamensis* (Sauvage, 1881)*, Pangasianodon gigas* Chevey, 1931*, P. hypophthalmus* (Sauvage, 1878)*, Pangasius bocourti* Sauvage, 1880*, P. larnaudii* Bocourt, 1866*, Probarbus jullieni* Sauvage, 1880*,* and *Puntioplites proctozysron* (Bleeker, 1865) using conventional PCR amplification and visualisation on a 1.5% agarose gel stained with SYBR® Safe–DNA Gel Stain (Life Technologies).

The qPCR assay was performed following the Osathanunkul & Minamoto^36,37^. A qPCR reaction of 20 µL included: 10.0 µL of TaqMan Environmental Master Mix 2.0 (Thermo Fisher Scientific), 2.0 µL of DNA template, 900 nM each of the F/R primers, and 125 nM of the probe. Reactions were run on a Qiagen Rotor gene Q thermal cycler with the following conditions: an initial step of 95 °C for 10 min, followed by 50 cycles of 95 °C for 15 s and 60 °C for 1 min. The standard curve for Siamese bat catfish was generated with serial dilutions series of synthesised target gene fragment standards (gBlocks™ Gene Fragments, IDT) with known copy numbers (150,000, 15,000, 1,500, 150, 15, and 1.5 copies per reaction with 12 technical replicates used for each of the dilution steps) as template for qPCR. The standard curve (y =  − 3.252x + 43.157; *R*^2^ = 0.99, efficiency = 103.00%) was generated using a 2 μL DNA template in a total reaction volume of 20 μL. All field samples were quantified in triplicate (three technical replicates). The average Cq across technical replicates was used for quantification. To determine an LOD and LOQ, curve-fitting model approach of Klymus et al.^38^ was used according to their published R script. As recommended by MIQE guidelines^39^, a Cq cut-off value was used to distinguish a positive signal from background based on the LOD for the qPCR assay^40–42^.

### PCR inhibition test

The inhibition of the PCRs of water samples was performed as described in previous study^43^. Primers and probe targeting the 16S rRNA of jellyfish species, *Chiropsoides buitendijki*, a marine species which does not inhabit the streams, were used to test for inhibition as internal controls (forward primer: 5′-CCCCAATCGAAATTAAGTTAGCC-3′; reverse primer: 5′-CACAGGTAGAGTGGAGAAATAGAG-3′; probe: 5′-FAM-GTGAAGACGCAGCTTTGTCT-TAMRA-3′). The oligo synthesis of *C. buitendijki* (1.5 × 10^2^ copies) was added to the samples (gBlocks™ Gene Fragments, IDT). ΔCq or Cq shift of ≥ 3 cycles of the internal controls in the water sample from the IPC in negative controls indicates of inhibition^44^.

### Statistical analysis

Linear regression is employed to elucidate the correlation between the quantity of rainfall and the detection of eDNA at individual sampling sites. The studies were conducted via the linear regression function and visualised using the R programming language. The eDNA detected on days without rain, which will be referred to as the initial eDNA concentration in this investigation, was classified into three unique groups according to their levels of concentration: *high* (when eDNA concentration exceeded 10 copies/mL), *moderate* (when eDNA concentration ranged from 10 to 3.0 copies/mL), *low* (when eDNA concentration ranged from 2.99 to 1.0 copies/mL), and *very low* (when eDNA concentration was below 1.0 copies/mL). Consequently, three sites (1, 2, and 4) were classified as exhibiting high concentration, while two sites (3 and 12) were classified as displaying moderate concentration. The remaining sites were designated as having low concentrations. Furthermore, the Thai Meteorological Department employed a classification system to categorise rainfall into five distinct groups based on specific criteria. These categories are outlined as follows: The term "*trace*" is used to describe rainfall volume that is below 0.1 mm. Rainfall between 0.1 and 10.0 mm is classified as "*light rain*," while rainfall ranging from 10.1 to 35.0 mm is classified as "*moderate rain*." "*Heavy rain*" refers to rainfall between 35.1 and 90.0 mm, while rainfall above 90.1 mm is referred to as "*very heavy rain*."

## Ethics statement

The present work received approval from the Animal Care and Use Committee evaluation at the Laboratory Animal Centre of Chiang Mai University, with protocol number 2561/FA-0001. The research was carried out with the permit of the Department of National Parks, Wildlife and Plant Conservation, Ministry of Natural Resources and Environment, Thailand, under DNP Permit No. 0907.4/18262, and the Department of Fisheries, Ministry of Agriculture and Cooperatives, Thailand, under DOF Permit No. 0510.5/3686. All methodologies were executed in adherence to the pertinent guidelines and legislation, in accordance with the suggestions outlined in the ARRIVE guidelines.

### Supplementary Information

Supplementary Information.

## Data Availability

The datasets generated and/or analysed during the current study are available in the GenBank repository, [Accession Numbers were provided in Supplementary Table S4]**.**
