# Supplementary material for: A comparative study on eDNA-based detection of Siamese bat catfish (Oreoglanis siamensis) in wet and dry conditions
Source: Sci Rep. 2024 Apr 17;14:8885. doi: 10.1038/s41598-024-58752-x (PMC11024149; doi:10.1038/s41598-024-58752-x)
Supplement: Supplementary file 1 — Supplementary Information. [file 41598_2024_58752_MOESM1_ESM.docx]

**Supplementary Materials for**

**A Comparative Study on eDNA-Based Detection of Siamese Bat Catfish (*Oreoglanis siamensis*) in Wet and Dry Conditions**

Maslin Osathanunkul ^1*^ and Chatmongkon Suwannapoom ^2*^

^1^ Department of Biology, Faculty of Science, Chiang Mai University, Muang District, Chiang Mai, Thailand

^2^ School of Agriculture and Natural Resources, University of Phayao, Muang District, Phayao, Thailand

Corresponding Author:

Maslin Osathanunkul*

Email address: maslin.cmu@gmail.com

Chatmongkon Suwannapoom*

Email address: chatmongkonup@gmail.com

**This PDF file includes:**

Supplementary Figures 1 to 2

Supplementary Table 1 to 4

**
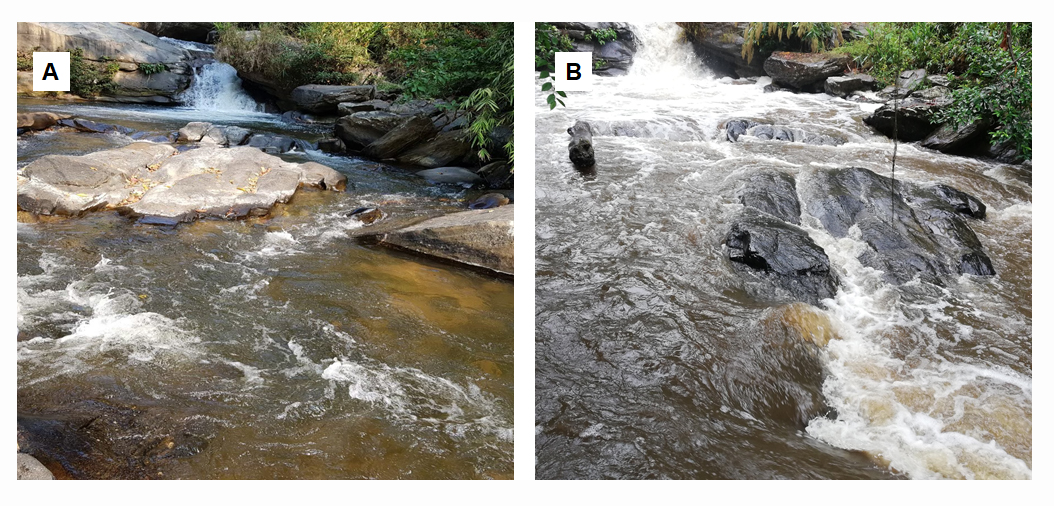
**

**Supplementary Figure 1**. A location where sampling took place on non-rainy (A) and rainy (B) days.

**
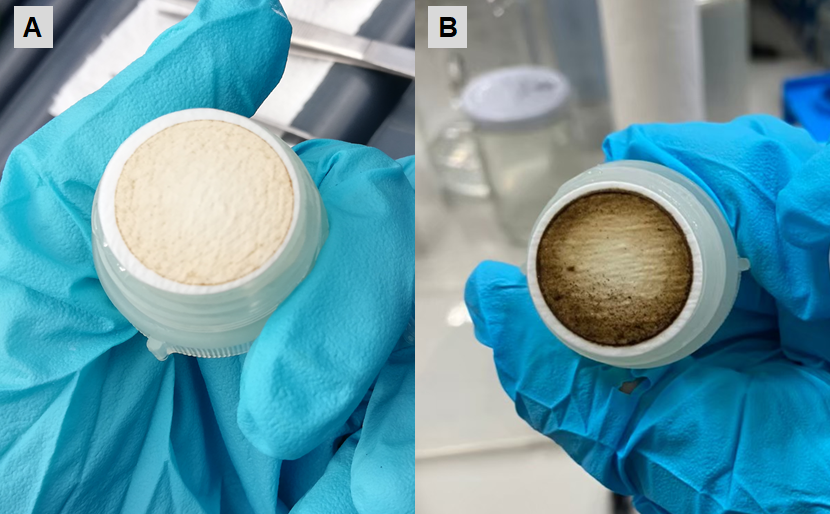
**

**Supplementary Figure 2.** Colour of the filtered papers at site 1 in two different weather conditions: (A) a non-rainy day and (B) a rainy day.

**Supplementary Table 1.** The eDNA concentration of Siamese bat catfish was observed in all three water replicates at each site during every sampling day, alongside the accompanying rainfall data. All qPCR results were reported into three categories which are (1) positive with quantifiable eDNA concentration, below limit of quantification *(bq*: Cq = 39.01 - 44.99), and non-detect (*nd*: Cq ≥ 45 or No amplification).

| **Sampling date** | | | **14 Dec 19** | **10 Feb 20** | **28 Jun 20** | **9 Sep 20** | **4 Oct 20** | **22 Nov 20** | **6 Feb 21** | **23 Mar 21** | **6 Apr 21** | **10 May 21** | **28 Aug 21** | **19 Nov 21** | **20 Feb 22** | **2 Mar 22** | **21 May 22** | **27 Aug 22** | **10 Sep 22** | **1 Oct 22** |
| --- | --- | --- | --- | --- | --- | --- | --- | --- | --- | --- | --- | --- | --- | --- | --- | --- | --- | --- | --- | --- |
| **Average rainfall (mm) / category** | | | **0 / *no rain*** | **0 / *no rain*** | **37 / *heavy*** | **46 / heavy** | **4 / *light*** | **0 / *no rain*** | **0 / *no rain*** | **3 / *light*** | **56 / heavy** | **5 / *light*** | **18 / *moderate*** | **0 / *no rain*** | **17 / *moderate*** | **0 / *no rain*** | **128 / very heavy** | **19 / *moderate*** | **88 / heavy** | **76 / heavy** |
| **Site 1** | **Average eDNA (copies/mL)** | **sample 1** | 19.64 | 20.36 | *—* | *—* | 19.24 | 20.92 | 20.57 | 18.94 | *—* | 18.13 | 14.97 | 20.63 | 15.27 | 20.98 | *—* | 14.27 | *—* | *—* |
|  |  | **sample 2** | 19.83 | 20.64 | *bq* | *bq* | 18.95 | 19.96 | 20.39 | 18.62 | *—* | 18.09 | 15.12 | 20.25 | 14.75 | 21.05 | *—* | 14.19 | *—* | *—* |
|  |  | **sample 3** | 20.57 | 21.06 | *—* | *bq* | 19.22 | 20.78 | 20.61 | 19.61 | *bq* | 18.36 | 15.33 | 20.70 | 15.51 | 21.21 | *—* | 14.92 | *—* | *—* |
|  |  | **Mean** | **20.01** | **20.69** | ***—*** | ***—*** | **19.14** | **20.55** | **20.52** | **19.06** | ***—*** | **18.19** | **15.14** | **20.53** | **15.18** | **21.08** | ***—*** | **14.46** | ***—*** | ***—*** |
|  |  | **SD** | 0.49 | 0.35 | *—* | *—* | 0.16 | 0.52 | 0.12 | 0.51 | *—* | 0.15 | 0.18 | 0.24 | 0.39 | 0.12 | *—* | 0.40 | *—* | *—* |
| **Site 2** | **Average eDNA (copies/mL)** | **sample 1** | 17.69 | 17.26 | *—* | *bq* | 16.15 | 17.83 | 17.37 | 8.94 | *bq* | 15.31 | 9.98 | 17.75 | 9.5 | 17.84 | *—* | 9.18 | *—* | *—* |
|  |  | **sample 2** | 17.73 | 17.31 | *bq* | *—* | 16.09 | 17.91 | 17.69 | 9.07 | *bq* | 15.08 | 10.26 | 17.54 | 9.41 | 17.97 | *—* | 9.06 | *—* | *—* |
|  |  | **sample 3** | 17.61 | 17.47 | *—* | *bq* | 16.18 | 18.03 | 17.51 | 9.16 | *—* | 15.17 | 10.17 | 17.29 | 9.63 | 18.13 | *—* | 9.15 | *—* | *—* |
|  |  | **Mean** | **17.68** | **17.35** | ***—*** | ***—*** | **16.14** | **17.92** | **17.52** | **9.06** | ***—*** | **15.19** | **10.14** | **17.53** | **9.51** | **17.98** | ***—*** | **9.13** | ***—*** | ***—*** |
|  |  | **SD** | 0.06 | 0.11 | *—* | *—* | 0.05 | 0.10 | 0.16 | 0.11 | *—* | 0.12 | 0.14 | 0.23 | 0.11 | 0.15 | *—* | 0.06 | *—* | *—* |
| **Site 3** | **Average eDNA (copies/mL)** | **sample 1** | 3.37 | 2.80 | *—* | *—* | *bq* | 3.07 | 2.84 | 1.43 | *—* | 0.98 | 0.51 | 0.97 | *bq* | 1.22 | *—* | *bq* | *—* | *—* |
|  |  | **sample 2** | 3.14 | 2.84 | *—* | *—* | 1.14 | 3.37 | 3.11 | *bq* | *—* | 1.01 | *bq* | 1.08 | 0.89 | 1.49 | *—* | 0.72 | *—* | *—* |
|  |  | **sample 3** | 3.57 | 2.56 | *—* | *—* | 1.02 | 3.27 | 3.06 | 1.18 | *—* | *bq* | 0.52 | 1.26 | 0.44 | 1.17 | *—* | 0.38 | *—* | *—* |
|  |  | **Mean** | **3.36** | **2.73** | ***—*** | ***—*** | **1.08** | **3.24** | **3.00** | **1.31** | ***—*** | **1.00** | **0.52** | **1.10** | **0.67** | **1.29** | ***—*** | **0.55** | ***—*** | ***—*** |
|  |  | **SD** | 0.21 | 0.15 | *—* | *—* | 0.08 | 0.15 | 0.14 | 0.18 | *—* | 0.02 | 0.01 | 0.15 | 0.32 | 0.17 | *—* | 0.24 | *—* | *—* |
| **Site 4** | **Average eDNA (copies/mL)** | **sample 1** | 12.57 | 11.62 | *—* | *—* | 10.25 | 12.01 | 12.31 | 9.41 | *—* | 9.41 | 6.62 | 11.97 | 5.96 | 12.55 | *—* | 5.62 | *—* | *—* |
|  |  | **sample 2** | 12.47 | 11.81 | *—* | *—* | 10.62 | 12.44 | 12.19 | 10.18 | *—* | 9.95 | 6.88 | 12.09 | 5.84 | 12.04 | *—* | 5.89 | *—* | *—* |
|  |  | **sample 3** | 12.24 | 12.00 | *—* | *—* | 10.44 | 12.31 | 12.42 | 9.73 | *—* | 9.68 | 7.04 | 11.91 | 5.79 | 12.27 | *—* | 5.32 | *—* | *—* |
|  |  | **Mean** | **12.43** | **11.81** | ***—*** | ***—*** | **10.44** | **12.25** | **12.31** | **9.77** | ***—*** | **9.68** | **6.85** | **11.99** | **5.86** | **12.29** | ***—*** | **5.61** | ***—*** | ***—*** |
|  |  | **SD** | 0.17 | 0.19 | *—* | *—* | 0.19 | 0.22 | 0.12 | 0.39 | *—* | 0.27 | 0.21 | 0.09 | 0.09 | 0.26 | *—* | 0.29 | *—* | *—* |
| **Site 5** | **Average eDNA (copies/mL)** | **sample 1** | *bq* | *bq* | *—* | *—* | 0.48 | 0.94 | 0.95 | *bq* | *—* | *bq* | *—* | 1.59 | *—* | 1.94 | *—* | *—* | *—* | *—* |
|  |  | **sample 2** | 1.25 | 1.48 | *—* | *—* | *bq* | 1.01 | 1.51 | 0.51 | *—* | 0.57 | *—* | 1.45 | *—* | 2.02 | *—* | *—* | *—* | *—* |
|  |  | **sample 3** | 1.44 | 1.22 | *—* | *—* | 0.50 | *bq* | 1.32 | 0.49 | *—* | 0.53 | *—* | 1.67 | *—* | 1.53 | *—* | *—* | *—* | *—* |
|  |  | **Mean** | **1.34** | **1.35** | ***—*** | ***—*** | **0.49** | **0.97** | **1.26** | **0.50** | ***—*** | **0.55** | ***—*** | **1.57** | ***—*** | **1.83** | ***—*** | ***—*** | ***—*** | ***—*** |
|  |  | **SD** | 0.13 | 0.19 | *—* | *—* | 0.01 | 0.05 | 0.28 | 0.01 | *—* | 0.03 | *—* | 0.11 | *—* | 0.26 | *—* | *—* | *—* | *—* |
| **Site 6** | **Average eDNA (copies/mL)** | **sample 1** | *bq* | 1.71 | *—* | *—* | *bq* | 1.52 | 2.01 | *bq* | *—* | *—* | *—* | 0.79 | *—* | *bq* | *—* | *—* | *—* | *—* |
|  |  | **sample 2** | 1.75 | 1.46 | *—* | *—* | 0.52 | 1.29 | *bq* | 0.56 | *—* | *—* | *—* | 0.61 | *—* | 1.02 | *—* | *—* | *—* | *—* |
|  |  | **sample 3** | 1.52 | *bq* | *—* | *—* | 0.49 | *bq* | 1.91 | 0.71 | *—* | *—* | *—* | *bq* | *—* | 0.72 | *—* | *—* | *—* | *—* |
|  |  | **Mean** | **1.64** | **1.59** | ***—*** | ***—*** | **0.51** | **1.41** | **1.96** | **0.64** | ***—*** | ***—*** | ***—*** | **0.70** | ***—*** | **0.87** | ***—*** | ***—*** | ***—*** | ***—*** |
|  |  | **SD** | 0.16 | 0.18 | *—* | *—* | 0.02 | 0.16 | 0.07 | 0.11 | *—* | *—* | *—* | 0.13 | *—* | 0.21 | *—* | *—* | *—* | *—* |
| **Site 7** | **Average eDNA (copies/mL)** | **sample 1** | 1.08 | *bq* | *—* | *—* | *bq* | 1.33 | 1.38 | 0.64 | *—* | *bq* | *—* | 0.95 | *—* | 1.13 | *—* | *—* | *—* | *—* |
|  |  | **sample 2** | 0.99 | 1.38 | *—* | *—* | 0.53 | *bq* | 1.07 | 0.52 | *—* | 0.59 | *—* | 1.15 | *—* | 1.07 | *—* | *—* | *—* | *—* |
|  |  | **sample 3** | *bq* | 1.05 | *—* | *—* | 0.50 | 1.07 | *bq* | *bq* | *—* | 0.51 | *—* | 1.26 | *—* | 0.94 | *—* | *—* | *—* | *—* |
|  |  | **Mean** | **1.04** | **1.21** | ***—*** | ***—*** | **0.52** | **1.20** | **1.23** | **0.58** | ***—*** | **0.55** | ***—*** | **1.12** | ***—*** | **1.05** | ***—*** | ***—*** | ***—*** | ***—*** |
|  |  | **SD** | 0.06 | 0.23 | *—* | *—* | 0.02 | 0.18 | 0.22 | 0.08 | *—* | 0.06 | *—* | 0.16 | *—* | 0.10 | *—* | *—* | *—* | *—* |
| **Site 8** | **Average eDNA* (copies/mL)** | **sample 1** | 1.93 | 2.11 | *—* | *—* | *bq* | 2.14 | 2.41 | 1.24 | *—* | *bq* | *—* | 1.95 | *—* | 2.34 | *—* | *—* | *—* | *—* |
|  |  | **sample 2** | 2.12 | 2.34 | *—* | *—* | 1.17 | 2.54 | 2.17 | 0.97 | *—* | 1.25 | *—* | 2.15 | *—* | 2.76 | *—* | *—* | *—* | *—* |
|  |  | **sample 3** | 2.46 | 2.09 | *—* | *—* | 0.94 | 2.43 | 2.07 | *bq* | *—* | 0.87 | *—* | 2.43 | *—* | 2.17 | *—* | *—* | *—* | *—* |
|  |  | **Mean** | **2.17** | **2.18** | ***—*** | ***—*** | **1.06** | **2.37** | **2.22** | **1.11** | ***—*** | **1.06** | ***—*** | **2.18** | ***—*** | **2.42** | ***—*** | ***—*** | ***—*** | ***—*** |
|  |  | **SD** | 0.27 | 0.14 | *—* | *—* | 0.16 | 0.21 | 0.17 | 0.19 | *—* | 0.27 | *—* | 0.24 | *—* | 0.30 | *—* | *—* | *—* | *—* |
| **Site 9** | **Average eDNA (copies/mL)** | **sample 1** | 1.65 | 2.25 | *—* | *—* | *bq* | 2.15 | 2.28 | *bq* | *—* | *bq* | *—* | 1.93 | *—* | 2.35 | *—* | *—* | *—* | *—* |
|  |  | **sample 2** | 1.83 | 1.81 | *—* | *—* | 0.86 | 1.89 | 2.08 | 1.02 | *—* | *bq* | *—* | 2.28 | *—* | 2.04 | *—* | *—* | *—* | *—* |
|  |  | **sample 3** | 1.94 | 2.00 | *—* | *—* | 0.71 | 2.31 | 1.84 | 0.81 | *—* | *bq* | *—* | 2.01 | *—* | 2.11 | *—* | *—* | *—* | *—* |
|  |  | **Mean** | **1.81** | **2.02** | ***—*** | ***—*** | **0.79** | **2.12** | **2.07** | **0.92** | ***—*** | ***—*** | ***—*** | **2.07** | ***—*** | **2.17** | ***—*** | ***—*** | ***—*** | ***—*** |
|  |  | **SD** | 0.15 | 0.22 | *—* | *—* | 0.11 | 0.21 | 0.22 | 0.15 | *—* | *—* | *—* | 0.18 | *—* | 0.16 | *—* | *—* | *—* | *—* |
| **Site 10** | **Average eDNA (copies/mL)** | **sample 1** | 2.21 | 1.87 | *—* | *—* | *bq* | 2.54 | 2.25 | 0.83 | *—* | 0.73 | *—* | 2.11 | *—* | 2.34 | *—* | *—* | *—* | *—* |
|  |  | **sample 2** | 2.01 | 2.17 | *—* | *—* | 0.98 | 2.10 | 2.05 | 1.02 | *—* | 0.99 | *—* | 2.17 | *—* | 2.05 | *—* | *—* | *—* | *—* |
|  |  | **sample 3** | 2.07 | 2.05 | *—* | *—* | 0.83 | 2.03 | 2.07 | *bq* | *—* | *bq* | *—* | 1.97 | *—* | 2.11 | *—* | *—* | *—* | *—* |
|  |  | **Mean** | **2.10** | **2.03** | ***—*** | ***—*** | **0.91** | **2.22** | **2.12** | **0.93** | ***—*** | **0.86** | ***—*** | **2.08** | ***—*** | **2.17** | ***—*** | ***—*** | ***—*** | ***—*** |
|  |  | **SD** | 0.10 | 0.15 | *—* | *—* | 0.11 | 0.28 | 0.11 | 0.13 | *—* | 0.18 | *—* | 0.10 | *—* | 0.15 | *—* | *—* | *—* | *—* |
| **Site 11** | **Average eDNA (copies/mL)** | **sample 1** | 1.85 | 1.94 | *—* | *—* | 0.93 | 2.01 | 1.95 | 0.89 | *—* | 2.04 | *—* | 2.12 | *—* | 2.08 | *—* | *—* | *—* | *—* |
|  |  | **sample 2** | 2.15 | 2.11 | *—* | *—* | 1.24 | 2.38 | 2.12 | 1.05 | *—* | 2.39 | *—* | 2.33 | *—* | 2.46 | *—* | *—* | *—* | *—* |
|  |  | **sample 3** | 2.08 | 2.29 | *—* | *—* | 1.11 | 2.08 | 2.24 | *bq* | *—* | 2.21 | *—* | 2.18 | *—* | 2.21 | *—* | *—* | *—* | *—* |
|  |  | **Mean** | **2.03** | **2.11** | ***—*** | ***—*** | **1.09** | **2.16** | **2.10** | **0.97** | ***—*** | **2.21** | ***—*** | **2.21** | ***—*** | **2.25** | ***—*** | ***—*** | ***—*** | ***—*** |
|  |  | **SD** | 0.16 | 0.18 | *—* | *—* | 0.16 | 0.20 | 0.15 | 0.11 | *—* | 0.18 | *—* | 0.11 | *—* | 0.19 | *—* | *—* | *—* | *—* |
| **Site 12** | **Average eDNA (copies/mL)** | **sample 1** | 4.12 | 4.01 | *—* | *—* | 1.82 | 3.98 | 4.15 | 1.86 | *—* | 1.97 | 0.95 | — | *bq* | 4.32 | *—* | *bq* | *—* | *—* |
|  |  | **sample 2** | 4.45 | 4.12 | *—* | *—* | 1.93 | 4.06 | 4.35 | 2.05 | *—* | 1.9 | *bq* | — | 1.01 | 4.12 | *—* | 0.85 | *—* | *—* |
|  |  | **sample 3** | 4.31 | 4.23 | *—* | *—* | 1.64 | 3.89 | 4.05 | 1.87 | *—* | 1.68 | 1.16 | — | 1.15 | 3.93 | *—* | 1.19 | *—* | *—* |
|  |  | **Mean** | **4.29** | **4.12** | ***—*** | ***—*** | **1.80** | **3.98** | **4.18** | **1.93** | ***—*** | **1.85** | **1.06** | ***—*** | **1.08** | **4.12** | ***—*** | **1.02** | ***—*** | ***—*** |
|  |  | **SD** | 0.17 | 0.11 | *—* | *—* | 0.15 | 0.09 | 0.15 | 0.11 | *—* | 0.15 | 0.15 | *—* | 0.10 | 0.20 | *—* | 0.24 | *—* | *—* |

**Supplementary Table 2.** Outcomes obtained from a linear regression analysis conducted to examine the association between the detection of eDNA and the amount of rainfall.

| **Site** | **eDNA detection level** | **Rain effect regression** | ***R^2^*** | ***p*-value** |
| --- | --- | --- | --- | --- |
| 1 | High | -0.47505 | 0.9165 | 2.853E-07 |
| 2 | High | -0.43531 | 0.9713 | 7.794E-10 |
| 3 | Moderate | -0.08238 | 0.9352 | 7.014E-08 |
| 4 | Low | -0.32469 | 0.9800 | 1.064E-10 |
| 5 | Low | -0.09078 | 0.8280 | 2.579E-04 |
| 6 | Low | -0.10117 | 0.8801 | 5.943E-05 |
| 7 | Low | -0.07093 | 0.8328 | 2.295E-04 |
| 8 | Low | -0.14030 | 0.8328 | 1.906E-04 |
| 9 | Low | -0.12357 | 0.8551 | 1.283E-04 |
| 10 | Low | -0.13334 | 0.8030 | 4.493E-04 |
| 11 | Low | -0.13126 | 0.8493 | 1.504E-04 |
| 12 | Moderate | -0.11357 | 0.9232 | 1.805E-07 |

**Supplementary Table 3.** Details of sampling sites chosen for this study.

| **Sites** | **Geographic coordinates** | **Remarks** |
| --- | --- | --- |
| 1 | 18.560556, 98.544889 | KP1 (Rodpai et al.^35^) |
| 2 | 18.560139, 98.545583 | KP2 (Rodpai et al.^35^) |
| 3 | 18.544389, 98.564500 | KP3 (Rodpai et al.^35^) |
| 4 | 18.541450, 98.572890 | KP4 (Rodpai et al.^35^) |
| 5 | 18.546560, 98.513250 | K1 (Rodpai et al.^35^) |
| 6 | 18.548260, 98.517550 | K2 (Rodpai et al.^35^) |
| 7 | 18.542000, 98.523180 | K4 (Rodpai et al.^35^) |
| 8 | 18.538390, 98.525160 | K5 (Rodpai et al.^35^) |
| 9 | 18.525210, 98.522250 | K7 (Rodpai et al.^35^) |
| 10 | 18.541880, 98.550110 | K8 (Rodpai et al.^35^) |
| 11 | 18.541690, 98.599690 | K9 (Rodpai et al.^35^) |
| 12 | 18.507180, 98.661670 | K10 (Rodpai et al.^35^) |

**Supplementary Table 4.** Accession number of COI sequences used in primer design

| **Species** | **Accession number** |  | **Species** | **Accession number** |
| --- | --- | --- | --- | --- |
| *Amblyceps foratum* | EU490875 |  | *Mystus bocourti* | EU490863 |
| *Bagarius bagarius* | MT812082 |  | *Mystus multiradiatus* | JX177677 |
| *Bagarius suchus* | DQ846698 |  | *Mystus singaringan* | MN992971 |
| *Bagarius yarrelli* | KM610424 |  | *Neolissochilus stracheyi* | MN096214 |
| *Bagrichthys majusculus* | - |  | *Notopterus notopterus* | JX983410 |
| *Bagrichthys obscurus* | - |  | *Opsarius koratensis* | HM224205 |
| *Barilius pulchellus* | HM224207 |  | *Opsarius pulchellus* | HM224207 |
| *Cephalocassis borneensis* | - |  | *Oreoglanis immaculatus* | JQ859840 |
| *Channa striata* | MW591031 |  | *Oreoglanis macropterus* | - |
| *Crossocheilus reticulatus* | MN342371 |  | *Oreoglanis siamensis* | MZ753673 |
| *Cyclocheilichthys repasson* | MW147376 |  | *Pareuchiloglanis anteanalis* | DQ508085 |
| *Danio albolineatus* | MN342400 |  | *Pareuchiloglanis feae* | - |
| *Devario aequipinnatus* | MT954943 |  | *Pareuchiloglanis hupingshanensis* | - |
| *Devario annandalei* | KY124375 |  | *Pareuchiloglanis myzostoma* | MH853829 |
| *Devario laoensis* | MF172771 |  | *Pareuchiloglanis sinensis* | MF122630 |
| *Discherodontus schroederi* | JX066749 |  | *Pethia stoliczkana* | MN342718 |
| *Esomus metallicus* | MW591064 |  | *Pseudomystus siamensis* | - |
| *Exostoma berdmorei* | DQ846699 |  | *Raiamas guttatus* | MK116352 |
| *Gyrinocheilus aymonieri* | MN342601 |  | *Rasbora paviana* | MW147436 |
| *Hemibagrus filamentus* | JQ289147 |  | *Scaphiodonichthys acanthopterus* | KJ994655 |
| *Hemibagrus spilopterus* | MK049455 |  | *Schistura poculi* | MG238142 |
| *Hemibagrus wyckii* | MG981082 |  | *Schistura pridii* | - |
| *Hemibagrus wyckioides* | EU490862 |  | *Systomus rubripinnis* | MK448198 |
| *Mastacembelus tinwini* | MW591093 |  | *Tor tambroides* | KU692919 |
| *Mystus albolineatus* | KF824812 |  |  |  |
